# Behavior and physiology of two different sow breeds in a farrowing environment during late 35-day lactation

**DOI:** 10.1371/journal.pone.0197152

**Published:** 2018-05-14

**Authors:** Honggui Liu, Ran Yi, Chao Wang, Peng Zhao, Mingyue Zhang, Shiwen Xu, Jun Bao

**Affiliations:** 1 College of Animal Science and Technology, Northeast Agricultural University, Harbin, P. R., China; 2 Key Laboratory of Swine Facilities Engineering, Ministry of Agriculture, Harbin, Hei Longjiang Province, China; 3 College of Veterinary Medicine, Northeast Agricultural University, Harbin, P. R., China; University of Missouri Columbia, UNITED STATES

## Abstract

To improve the overall welfare levels of sows and to reduce stress levels at late 35-day lactation, we selected targeted behavioral indicators that might be associated with stress. Therefore, we monitored and evaluated the adaptive capability of two different breeds of sows to the farrowing environment. In this study, Damin sows (Large White × Min pig sows, *n* = 20) and Large White sows (*n* = 20) were farrowed in individual pens. Saliva was collected and tested for cortisol density at –15 min, and then at +15, 30, 60, 90, 120, 180 and 240 min after an adrenocorticotropic hormone (ACTH) stimulation test conducted at 20, 27 and 34 d post-partum. The postures, including ventral and lateral recumbency to other postures, defecating, urinating, sham-chewing and bar-biting behavior, were observed by video from 07:00 to 09:00 and from 13:00 to 15:00 on the 7th day of each week from the 3rd to the 5th week post-parturition. In addition, the concentrations of salivary interleukin (IL)-6, tumor necrosis factor (TNF)-α and secretory immunoglobulin (SIgA) were assayed after the observed behaviors. The results showed no significant difference between Damin sows and Large White sows in terms of behaviors at the 3rd week. Additionally, there were no significant differences between Damin and Large White sows in terms of the behaviors of ventral recumbency and bar-biting with the exception of lateral recumbency to other postures, sham-chewing, defecation and urination in the fifth week. Meanwhlie, there was significant difference between two breeds in term of ventral recumbency at the 4th week. The result of the ACTH test showed a significant difference between the Damin and Large White sows by the 27th and 34th days postpartum (*P*<0.01). In addition, the serological concentrations of IL-6 and TNF-α were not significantly different between the two breeds at the 3rd week postpartum. However, these indicators were significantly different at the 5th week postpartum (*P* = 0.000, and *P* = 0.003, respectively). The SIgA concentrations in saliva were significantly different between breeds at the 3rd week postpartum (*P*<0.01). In conclusion, both breeds of sows maybe in a state of stress after the 4th week postpartum. However, the Damin sows may be better than the Large White sows in terms of adapting to this farrowing environment.

## Introduction

At present, in response to the concern of animal welfare advocates, there has been a strong interest in the loose farrowing environment of lactating sows [1]. However, in such an environment, the sows might gradually increase the stress experienced by piglets and the environment, especially during long lactation periods [2, 3]. Therefore, selecting adaptable breeds with the intention of reducing stress might further improve the welfare of lactating sows in late lactation, and could be an effective means of reducing the harm caused by a stressful environment. Therefore, we attempted to explore the behaviors of different breeds of sows to improve their adaptability and to decrease stress experienced over the course of 35 days by late lactating sows. For example, the Min pig is a local breed found in north-eastern China, and displays good maternal instincts. This is an outstanding characteristic of the Min pig and the Min pig hybrid breeds, which show resistance to stress [4–6]. Thus, by utilizing the above advantages, we selected primiparous Damin sows (Large White *×* Min pig sows) in an attempt to improve the adaptability of pigs in a farrowing environment during late lactation.

The behavior of a pig can reflect the interaction between its own biological requirements and its response to environmental conditions. Therefore, we can use behaviors to judge the adaptability of the animals to their environment. Some reports showed the behaviors of the sows, such as sham-chewing [7], bar-biting [8], excessive excretion [9–11] and other behaviors, might be related to a lack of adaptability to the environment [11]. To avoid the stress caused by the sow not being able to avoid the piglets attempt to stimulate milk let down by massaging the udder in late lactation, sows often show long standing and prone postures [5]. Therefore, here we used behavioral indicators, including ventral recumbency[12], lateral recumbency to other postures[12], defecation[12], urination[12], sham-chewing[12] and bar-biting[12], to evaluate an enriched environment during lactational stress, and also determined the degrees of adaptation and adjustment to the environment.

Changes in behaviors might be associated with changes in peripheral and central nervous system physiological processes [13, 14]. Therefore, we needed to monitor the endocrine secretions of sows. However, if one uses methods to determine urinary or fecal corticoid levels, these can be affected by social rank and experience during an environmental event (i.e., by stress or its duration). Therefore, these measured end-points will clearly cannot accurately reflect the state of chronic stress in any individual animal [2, 15–19]. In addition, due to the presence of glucocorticoid secretion and changes in the circadian rhythm, the hypothalamic–pituitary–adrenal axis can adapt to long-term stress durations. Thus, combining these measurements with assays of adrenocorticotropic hormone (ACTH) and the results of cortisol stimulation tests can be used to evaluate the chronic stress state of different breeds of sows [20].

The response of the immune system to stress is an adaptive mechanism showing how organisms have evolved to cope with challenging situations [21]. An insufficient adaptation can lead to reduced immune competence and increased disease susceptibility [21]. Thus, exploring the adaptability of the sows of two different breeds in the later period of lactation, we aimed to determine the concentration of secretory immunoglobulin (SIgA) in saliva [22, 23] and the levels of interleukin (IL)-6 and tumor necrosis factor (TNF)-α in serum [24]. This was necessary to expand our knowledge of the complex relationship between stressful events and immune functioning during late lactation. Moreover, there is growing interest in assessing psychosocial stress in farm animals to improve our understanding of their overall welfare and health.

## Materials and methods

### Ethics statement

All procedures used in the present study were approved by the Institutional Animal Care and Use Committee of Northeast Agricultural University (Approval No. IACECNEAU20121013).

### Animals

Forty sows were selected (20 Damin, and 20 Large White) that were mated with Duroc (Canadian) boars. Backfat values and weights of the two different breeds had no significant difference when mating at the age of 7–8 months (backfat:18.99 ± 1.22 vs 19.06 ± 0.91; weights: 113.39 ± 6.10 vs 115.99 ± 5.01 kg, respectively). They were fed in the same pens (5.7 m wide, and 4.5 m long, each containing 10 pregnant sows). The ground, of concrete, was covered with straw. There were three water sources and natural ventilation supplied for sows during pregnancy. Sows were randomly transferred to the parturition pen on the 7th day before the expected due date.

### Housing and management

**Fig 1:**
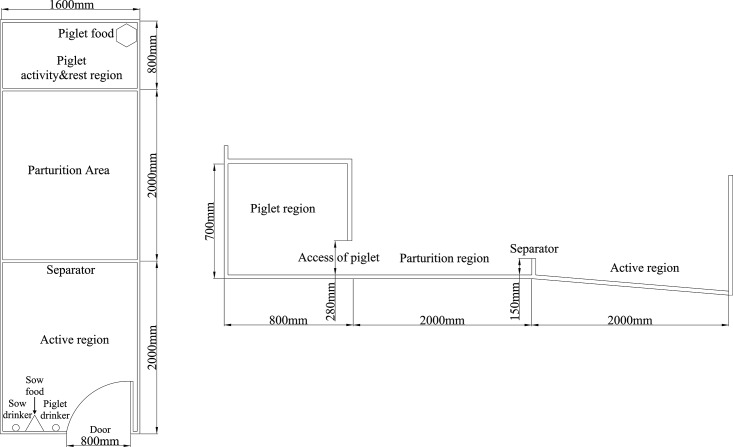
The design of the farrowing pens.

The design of the parturition pens is shown in Fig 1. The inside was 480 cm long, and 160 cm wide. The door of the parturition pen was 80 cm wide and 120 cm high. There was terrestrial heat in the piglet activity and rest region. It had access to the parturition area and only meet the piglet’s access freely. Meanwhile, the parturition area was also the activity and rest region for piglets. There was a low barrier in the door to prevent the piglets from leaving the rest areas during the first three days after birth. The sows could active freely. The feeding trough for piglets was 20 cm high and 60 cm wide. The ground inside the farrowing pens was of concrete, covered with straw (3.50 ± 0.25 kg). All of the straw was provided weekly. The sows were fed twice daily (at 07:30 and 16:00) in the farrowing pens. The health of the piglets was checked; the number of piglets experiencing diarrhea and swelling were recorded, and any sick piglets were removed at 06:30 each morning. Approximately 2 cm of bedding was added to the floor of the farrowing pens at 07:30 each weekend. These sows were fed 3 kg/day pre-parturition and were reduced by 0.5 kg/day 1 week before farrowing. On the first day after farrowing, the sows were fed 0.5 kg. Then, the ration on following days was increased by 0.5 kg/day until the sows could be fed *ad libitum*. After weaning, the rations of the primiparous sows were decreased to 3 kg/day until mating. All the sows were fed with complete feed containing the following constituents per kg: ME 12.9 MJ, crude protein 185.0 g, crude fat 50.0 g, crude ash 80.0 g, and lysine 12.0 g. During the study period, the temperature and relative humidity (both interior and exterior) of the farrowing pens were measured daily with a hygrothermograph (Kestrel 4000 Pocket Weather Tracker; Kestrel, Santa Cruz, CA, USA). The daily temperature and relative humidity inside the farrowing pens were measured at 08:00 (16.5°C; 90.0%), 14:00 (30.5°C; 30.4%) and 20:00 (21.6°C; 58.7%), in August and at 08:00 (8.7°C; 85.5%), 14:00 (33.0°C; 20.3%) and 20:00 (14.7°C; 51.3%) in September.

### Observations

#### Behavioral observations

The behaviors of the sows were recorded automatically by a video surveillance system (DS-IT5, Hangzhou Hikvision Digital Technology Co., Ltd., Hangzhou, P. R. China) for data acquisition to prevent artificially delimited observation times from impacting on the test results. From the 2nd to the 4th week and on the 3rd and 6th day each week post-parturition, all behaviors were recorded on video with sampling and continuous recording from 07:00 to 09:00, and 13:00 to 15:00. Data for continuous behaviors were recorded in numbers and converted into aggregate data over the total observation time. The parameters and their definitions are outlined in Table 1.

**Table 1:** Behavioral parameters and their definitions.

Behavior	Definition
**Ventral recumbency ^a^**	Sow’s chest and abdomen touching the floor and front legs stretched or folded under the body *
**Lateral recumbency to other postures ^b^**	Posture changing from lateral recumbency to other positions including ventral recumbency, sitting and standing*
**Defecation ^b^**	Elimination of feces from the body*
**Urination ^b^**	Discharge of urine from the body*
**Sham-chewing ^b^**	Chewing actions performed without the presence of food in the oral cavity*
**Bar-biting ^b^**	Stereotyped biting, gnawing, or sliding of the mouth on accessible part (usually metal bars) of an enclosure*

*Some behavioral parameters and their definitions are from Hurnik *et al*., (1995) [12]

^a^ These behaviors are presented as percentages of time.

^b^ These behaviors are presented as the hourly number of occurrences.

#### ACTH stimulation test

Synthetic ACTH (Ser–Tyr–Ser–Met–Glu–His–Phe–Arg–Trp–Gly–Lys–Pro–Val–Gly–Lys–Lys–Arg–Arg–Pro–Val–Lys–Val–Tyr–Pro), was diluted with 0.9% sterile saline, and injected into a marginal ear vein (dose of 10 pg/kg;[25]) (ACTH stimulation Test[20]). Saliva was collected and tested for the levels of cortisol at –15 min, and at 15, 30, 60, 90, 120, 180 and 240 min after the ACTH stimulation test at 20, 27 and 34 days postpartum[26].

#### Physiological measurements

Plasma concentrations of IL6 and TNF-α from 3rd to 5th week after parturition were measured using a commercial porcine enzyme-linked immunosorbent assay (ELISA) kits (Nanjing Jiancheng Bioengineering Institute, Nanjing, P. R. China) (Physiological measurements test[27]). Salivary concentrations of SIgA were assayed using a commercial porcine ELISA kit (Nanjing Jiancheng Bioengineering Institute).

### Statistical analyses

The study is a two-way crossed classification. All breed-dependent data was statistically analyzed with SPSS18.0. Normality test and homogeneity test were performed first. Data which were not normally distributed were log transformed using Transform (SPSS18.0). They were examined for normal distribution, considering skewness, kurtosis, the Shapiro–Wilks test for normality, and a normal probability plot. Statistical analysis method of LSD, SNK, Duncan and Bonferroni in Post-Hoc Teats in a GLM procedure of the SPSS statistical software (v. 18.0,) was used to test the behavioral differences between the two breeds in time-dependent observations, which found the following effects:

$$Y_{ijk}=\mu +{Breed}_{i}+{Time}_{j}+{Breed}_{i}\times {Time}_{j}+e_{ijk};$$

where *Y_ijk_* = value observed for characteristics analyzed; μ indicates the overall average; Breed_*i*_ indicates the effect of the breeds on behavior data; Time_*j*_ indicates the effect of observation weeks (W_3_, W_4_, W_5_) after parturition on behavior data; Breed_*i*_ × Time_*j*_ indicates the interaction between the breeds and the observed time; *e_ijk_* = random errors associated with observation. All data are presented as the mean ± standard deviation.

## Results

### Behavior

There was no significant difference in ventral recumbency behavior of the two breeds of sows at 3rd week postpartum (Table 2). However, at the 4th and 5th week, the rate of ventral recumbency was significantly different between breeds (F_0.05_(1,38) = 4.781; *P* = 0.035 and F_0.05_(1,38) = 6.551; *P* = 0.015, respectively). With extending the period of lactation, the two breeds of sows showed increasing ventral recumbency times, and that in *Large White* sows were significantly higher than that in Damin sows (F_0.05_(1,114) = 12,716; *P* = 0.001). There was no significant difference between the two breeds of sows from 3rd to 5th postpartum in terms of the behavior of lateral recumbency to other postures (F_0.05_(2,114) = 0.101; *P* = 0.101). With an extended period of lactation, both breeds gradually increased their frequency of the behavior of lateral recumbency to other postures, and that in *Large White* sows were significantly lower than that in Damin sows (F_0.05_(1,114) = 5.801; *P* = 0.018). In addition, with extending the period of lactation, the two breeds of sows showed increasing frequency of defecation, and that in *Large White* sows were significantly higher than that in Damin sows (F_0.05_(1,114) = 6.085; *P* = 0.015), and there were no significant differences in the frequency of urination or defecation between the breeds (P>0.05). Further, there were no significant differences between breeds in terms of sham-chewing (F_0.05_(1,114) = 1.086; *P* = 0.300). However, there was significant difference between the two breeds of sows from 3rd to 5th postpartum in terms of sham-chewing (F_0.05_(2,114) = 4.343; *P* = 0.015). With extending the period of lactation, the two breeds of sows showed increasing frequency of bar-biting, and that in *Large White* sows were significantly higher than that in Damin sows (*P*<0.01), and there was also significant difference between the two breeds of sows from 3rd to 5th postpartum in terms of bar-biting (*P*<0.01). All the detail data of behavioral parameters are respectively shown in Table 2.

**Table 2:** Behavioral differences and changes in both breeds of sows 3 weeks post-parturition in the same environmental conditions.

Behavioral parameters	Breeds	Times			Time effect
		3W	4W	5W	
**Ventral recumbency**	*Damin*	11.41^a^±2.41	12.72^bx^±4.56	14.79^cx^±2.98	P = 0.000; F_0.05_(2, 114) = 10.264
	*Large White*	13.06^a^±4.87	15.89^by^±4.61	18.35^cy^±5.45	
	*Breed effect*	P = 0.001; F_0.05_(1, 114) = 12.716			P = 0.575; F_0.05_(2, 114) = 0.557
**Lateral recumbency to other postures**	*Damin*	1.55±0.51	1.70±0.47	1.85±0.49	P = 0.101; F_0.05_(2, 114) = 0.101
	*Large White*	1.40±0.48	1.40±0.50	1.60±0.50	
	*Breed effect*	P = 0.018; F_0.05_(1, 114) = 5.801			P = 0.813; F_0.05_(2, 114) = 0.207
**Defecation**	*Damin*	0.80±0.41	0.85±0.37	0.95±0.22	P = 0.230; F_0.05_(2, 114) = 1.490
	*Large White*	0.95±0.22	1.00±0.00	1.00±0.00	
	*Breed effect*	P = 0.015; F_0.05_(1, 114) = 6.085			P = 0.610; F_0.05_(2, 114) = 0.497
**Urination**	*Damin*	0.95±0.22	0.95±0.22	0.90±0.31	P = 0.265; F_0.05_(2, 114) = 1.490
	*Large White*	0.95±0.22	1.00±0.00	0.85±0.49	
	*Breed effect*	P = 1.000; F_0.05_(1, 114) = 0.000			P = 0.734; F_0.05_(2, 114) = 0.310
**Sham-chewing**	*Damin*	0.85±0.37	1.00±0.00	1.00±0.00	P = 0.015; F_0.05_(2, 114) = 4.343
	*Large White*	0.95±0.22	1.00±0.00	1.00±0.00	
	*Breed effect*	P = 0.300; F_0.05_(1, 114) = 1.086			P = 0.341; F_0.05_(2, 114) = 1.086
**Bar-biting**	*Damin*	0.95±0.22	1.00±0.00	1.05^x^±0.22	P = 0.000; F_0.05_(2, 114) = 92.361
	*Large White*	0.85^a^±0.37	1.05^b^±0.22	2.00^cy^±0.00	
	*Breed effect*	P = 0.000; F_0.05_(1, 114) = 57.000			P = 0.000; F_0.05_(2, 114) = 68.083

Note: Different superscript letters in a row indicate significant differences between observation weeks (^a^ vs. ^b^ vs. ^c^ *P*<0.05).

Different superscript letters in a column indicate significant differences between breeds (^x^ vs. ^y^ *P*<0.05).

Means and standard deviation are shown.

### ACTH stimulation test

**Fig 2:**
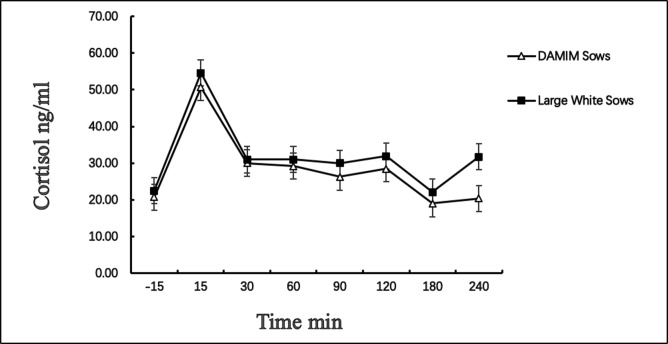
A tend of the changes in cortisol concentrations in saliva following the ACTH stimulation test at the 34th day postpartum. All data are presented as the mean ± standard deviation.

An ACTH stimulation test was conducted in both breeds of sows at the 20th, 27th and 34th days postpartum. There were no significant changes in the concentration of cortisol on the 20th day after farrowing. However, at 27th and 34th days postpartum, before the level of cortisol returned to a normal level, the salivary levels increased rapidly after the injection of ACTH. Cortisol concentrations in saliva were significantly higher in Large White sows than in Damin sows at each time point (*P*<0.05). In addition, there were similar results of testing at 34th day postpartum. A tend of the changes in cortisol concentrations in saliva following the ACTH stimulation test at the 34th day postpartum is shown in Fig 2.

### Physiological indexes

The serum levels of IL-6 in both breeds of sows were not significantly different at the 3rd week postpartum; however, the levels were significantly different at the 4th and 5th weeks (F_0.05_(1,38) = 5.045; *P* = 0.031 and F_0.05_(1,38) = 543.253; *P* = 0.000, respectively). The levels of TNF-α in serum in the two breeds of sows were not significantly different at the 3rd and 4th week postpartum. However, they were significantly different at week 5 (F_0.05_(1,38) = 10.055; *P* = 0.003). The SIgA concentrations in saliva in the two breeds of sows were significantly different at the 3rd and 5th weeks postpartum(F_0.05_(1,38) = 40.411; P = 0.000). With extension of the testing time, the TNF-α concentrations in serum decreased gradually in both breeds of sows. By contrast, the levels of IL-6 in serum in Damin sows tended to increase, while they increased first and then decreased in Large White sows. In addition, the SIgA concentration in saliva in two breeds of sows tended to decrease first, and then increase. The results are shown in Table 3.

**Table 3:** Effect of breeds on the physiological indexes of sows at different stages.

Physiological indexes	Breeds	Times			Time effect
		3W	4W	5W	
**IL6 (pg/ml)**	*Damin*	4.81^a^±1.12	5.09^ax^±0.22	6.34^bx^±0.18	P = 0.000; F_0.05_(2, 114) = 11.257
	*Large White*	4.45^a^±0.62	5.70^ay^±1.21	4.07^by^±0.40	
	*Breed effect*	P = 0.000; F_0.05_(1, 114) = 24.493			P = 0.000; F_0.05_(2, 114) = 38.953
**TNF-α (pg/ml)**	*Damin*	195.36^a^±18.02	171.87^b^±21.27	161.33^bx^±17.57	P = 0.000; F_0.05_(2, 114) = 47.874
	*Large White*	186.52^a^±14.03	163.94^b^±16.38	143.12^cy^±18.73	
	*Breed effect*	P = 0.000; F_0.05_(1, 114) = 12.868			P = 0.813; F_0.05_(2, 114) = 0.207
**SIgA (μg/ml)**	*Damin*	28.45^ax^±1.43	20.73^b^±2.66	24.12^c^±2.52	P = 0.000; F_0.05_(2, 114) = 65.642
	*Large White*	25.96^ay^±1.01	20.52^b^±3.65	23.70^c^±3.13	
	*Breed effect*	P = 0.028; F_0.05_(1, 114) = 4.955			P = 0.095; F_0.05_(2, 114) = 2.400

Note: Different superscripts in a column indicate significant differences between observation weeks (^x^ vs. ^y^ *P*<0.05).

Different superscripts in a row indicate significant differences between breeds. (^a^ vs. ^b^ vs. ^c^ *P*<0.05).

Means and standard deviations are shown.

## Discussion

### Behavior

The behaviors of ventral recumbency and lateral recumbency to other postures were two important signals indicating that sows refused to allow piglets to suckle [28, 29]. In this study, from the trend of ventral recumbency at 3–5 weeks postpartum, both breeds of sows gradually prevented piglets from stimulating the udder[30]. Thus, an enriched environment for farrowing might not necessarily eliminate this effect, because of the influence of both breeds of sows caused by extension of the time for breastfeeding piglets. However, the incidence of ventral recumbency was significantly lower in Damin sows than in Large White sows, instead, more behavior of lateral recumbency to other postures were shown in Damin sows. It may show that they may indicate more willingness to nurse, indicating that the former had certain genetic and biological advantages in response to late lactational stress as shown by the Min pig, including good motherhood[5].

Some authors believe that excessive excretion is linked with the animal’s negative emotions and stress [31]. Yet, in this experiment, there was no such excessive urination in the two breeds of sow and the result might be related to the enriched environment for the farrowing of piglets, given that both breeds of sows received reduced stress. However, such behaviors might be induced by the stress of serious environmental restraint; for example, sows in a limited space will exhibit excessive urination behavior [32]. In this study, we found the behavior of defecation was influenced by breeds all the time. We speculated that the result may be related to the feed intakes and excretion law of different breeds in the late lactation, and that was no correlation with stress conditions. It was required to do further specific research to determine the value of defecation behavior for evaluating the adaptation of sows in the late lactation.

From the perspective of adaptability, when the animals were in a poor environment, they would try to counteract adverse effects in various ways such as behavioral and physiological methods [33]. Some scholars believed that sham-chewing was one of the most common stereotypic behaviors of sows reared in the crate environment [34]. The occurrence of sham-chewing was caused by restricted feeding, and eating motivation caused by starvation increases to the shift of behavioral direction [8]. In this study, the main reason why there was no difference of sham-chewing between two breeds may be the loose farrowing environment and sufficient feeding in lactation condition could not lead to sham-chewing. The mentioned point above of view was in conformity with the actual result. The behaviors of and bar-biting at the 5th week postpartum were more prevalent in Large Whit*e* sows than in Damin sows. This showed that the former breed may exhibit a more obvious maladjustment in response to late lactation stress. This was similar to the conclusions made by others [32]. At the same time, we also believe that there is important value in studying abnormal behaviors such as and bar-biting in measuring the adaptability of lactating sows to a late lactating environment.

### Physiological indexes

The adrenal gland response to a challenge with ACTH is a measure of chronic physiological stress [35]. Adaptation of HPA axis to chronic stress involves increased adrenocortical response to ACTH, which increases the level of cortisol during acute stress stimulation. In this study, the level of salivary cortisol before ACTH stimulation test was used as the control to compare the changes of the level of salivary cortisol at different time points after ACTH stimulation test. ACTH stimulation test on the 20th day postpartum showed that there were no significant changes in the level of salivary cortisol, which might be due to the high stress of sows from the factors of both the environment and piglets. The results of ACTH stimulation test on day27 showed that the sows showed a response of stress at this time. And the stress response of ACTH stimulation test still showed on day34. The results showed that chronic stress was still evident in both breeds of sows, despite them living in enriched environmental farrowing pens at the 5th week postpartum. Compared with day20, the ability of resisting the stress of sows obviously decreased on day27 and day34, and they were at the level of low stress. We speculate that the sows were in the chronic stress. Cronin et al. (1991) [36]and Jarvis et al. (2006) [37]suggest that during longer lactation, when sows begin to show signs of chronic stress, it may be harmful to sows. This was the farm manager’s set time for weaning of piglets and provided a theoretical basis for enhancing the welfare levels of the sows. In addition, ACTH stimulation test results showed that salivary cortisol concentrations were higher in Large White sows than in Damin sows, and the main reason for this difference might be breed-related.

It is a natural tendency to spend time away from their piglets as lactation progresses and gradually reduce suckling frequency, effectively creating a gradual weaning process [38]. When the frequency of lactation cannot be reduced, the external environment and piglets are likely to produce stress on sows, which is not conducive to the welfare of sows. Some researchers speculate that sows may adapt to the chronic effects produced by environment and piglets during lactation, and the response of the stress system may be reduced to a level indistinguishable from that of non-stressed individuals [39]. The Damin and Large White sows showed significant differences in the levels of IL-6 in serum at the 4th and 5th weeks postpartum, and Damin sows showed higher levels than Large White sows at the 5th week postpartum. We believe a major reason for this difference might arise from the pro- and anti-inflammatory properties of IL-6 [40]. In addition, the insensitivity of plasma IL-6 levels to stress was reduced, and this might be associated with the anti-inflammatory action of this cytokine. At the 5th week, Damin sows adapted better to the late lactation environment by increasing a compensatory and mediating immunoregulation with beneficial cytokine secretion [41, 42]. However, the adaptability to the stress of late lactation of Large White sows decreased in the 5th week, and decreases in the levels of IL-6 were observed. The reluctance for lactation that was shown at the 5th week of the stress environment and experienced by Large White sows might pose a threat to their health. Thus, in the current study, the changed levels of IL-6 showed that the adaptability of Damin sows during late lactation was stronger than that of Large White sows. Also, with prolonged lactation, levels of IL-6 in serum in the Damin sows increased significantly. Moreover, the levels of IL-6 in serum in Large White sows increased first, and then decreased. Our observation showed that the levels of IL-6 in serum decreased or remained unchanged, but that finding is inconsistent with other studies [21, 43]. The reasons might have been related to the different breeds of sows employed in the studies. In addition, changes in cell distribution might reflect psychological effects of the stressor, which would be characterized by an uncontrolled and unpredictable response to environmental conditions with a display of anxiety [44–46].

Experiments conducted with Damin sows during lactation at the 5th week showed that TNF-α concentrations in serum were higher than those found in Large White sows. We believe that the main reasons for the observed differences in behavior might be due to the action of this cytokine. This anti-inflammatory factor played a key role in the response of Damin sows to the late lactation environment and a marked participation in Large White sows. However, with an extended duration of lactation, the serum TNF-α levels decreased gradually in both breeds of sows under stress conditions, similar to previous reports [43, 47–49]. A possible explanation for the stress-induced modulation of TNF-α production might be due to an adaptive response that can initiate enhanced immune surveillance capable of preparing the animal for potential mediators of danger [50, 51]. Thus, our findings emphasize the particular importance of TNF-α in mediating the adaptive response to short-term psychosocial stress in sows.

We found that the levels of SIgA in saliva increased with the duration of lactation, and this was discordant with previously published work [22]. We believe that the levels of SIgA in saliva may represent a transient index of the emotional state at the time that the test pigs were assessed.

## Conclusions

Min pig hybrids in late lactation remained susceptible to chronic stress. However, in response to chronic stress behavior and immune responses, the Large White sows adapted better to the environment of late lactation, and clearly this adaptive capacity differed between the two breeds of sows. At the same time, the results of actual production might improve the welfare of sows and determine the weaning age of piglets. There might be benefits from additional research and field studies in this field.

## Supporting information

S1 Dataset The Data of behavior and physiological indexes. (XLSX)

S2 Dataset The data of ACTH test. (XLS)
